# Inherited Thrombophilia and Recurrent Pregnancy Loss

**DOI:** 10.5812/ircmj.13708

**Published:** 2013-12-05

**Authors:** Alireza Parand, Jale Zolghadri, Mozhgan Nezam, Abdolreza Afrasiabi, Sezaneh Haghpanah, Mehran Karimi

**Affiliations:** 1 Iranian Hospital, Dubai, UAE; 2 Infertility Research Center, Gynecology and Obstetrics Department, Shiraz University of Medical Sciences, Shiraz, IR Iran; 3 Hematology Research Center, Shiraz University of Medical Sciences, Shiraz, IR Iran

**Keywords:** Inherited, Pregnancy loss, Thrombophilia

## Abstract

**Background:**

Recurrent pregnancy loss (RPL) is a common health problem. The polymorphisms G20210A of prothrombin gene (FII G 20210A), and G 1691A of factor V gene (Factor V Leiden, FVL) are the most extensively studied thrombophilic mutations in association to recurrent miscarriage.

**Objectives:**

To determine the frequency of FII G20210A and FVL polymorphisms as well as protein C and protein S deficiency in a series of patients with RPL compared with control group.

**Patients and Methods:**

The study group included 90 randomly selected patients with three or more consecutive miscarriages with the same partner in <20 weeks gestation in 2012. The control population consisted of 44 age-matched women with at least one live born children and no history of pregnancy loss. Functional activity of protein C and S, activated protein C resistance, FVL assay by polymerase chain reaction and prothrombin gene mutation were assessed. The polymorphism frequencies were recorded for each group and comparisons were made.

**Results:**

The mean functional activity of protein C and protein S were not significantly different between case and control groups (P >0.05). Frequency of protein C deficiency was also not significantly different between the case and control groups (P=0.906), but frequency of protein S deficiency was significantly higher in patients than controls (P=0.03). Genotype pattern of the patients and healthy individuals were not significantly different with regard to either FVL or Prothrombin G20210A (P > 0.05).

**Conclusions:**

We determined a significant higher frequency of protein S deficiency in patients with RPL compared with controls. But the frequency of protein C deficiency and the frequency of two common thrombophilic mutations (Factor V Leiden and Prothrombin G20210A), were not significantly different between patients with recurrent miscarriage and healthy women.

## 1. Background

Prothrombotic disorders have been associated with the pathophysiology of many obstetric complications of placental origin (e.g. stillbirth, fetal growth restriction, severe preeclampsia and placental abruption) and congenital thrombophilia had inevitably drawn the scientific interest for its potential link recurrent miscarriage. Recurrent pregnancy loss (RPL) is a common health problem, with three or more loses affecting 1-2% and two or more losses affecting up to 5% of women at the reproductive age (1, 2).

The polymorphisms G20210A of prothrombin gene (FII G 20210A) and G 1691A of factor V gene (Factor V Leiden, FVL) and C677T of methylene tetrahydrofolate reductase gene (MTHFR C677T) are the most extensively studied thrombophilic mutations in association to recurrent miscarriage. It appears that the presence of FII G20210A and Factor V Leiden mutation increases the risk for recurrent early pregnancy loss (odds ratios 2.49 for FII G20210A, 2.71 for homozygous and 1.68 for heterozygous FVL), whereas homozygosity for MTHFR C677F is not significantly increasing the risk (odds ratio 1.40, 95% CI 0.77-2.55) (3).

The R2 haplotype of factor V is characterized by a mild reduction of total factor V levels, with a relative increase of the more thrombogenic isoform, FVL (4). The polymorphism Arg1299His (A 4070G) is one of the mutations resulting in the R2 haplotype, and its association with thrombotic events has been variable (5-7). In contrast to FVL, the presence of A1299H does not appear to increase the risk for recurrent miscarriage.

A second common mutation for the MTHFR gene is produced by an A to C transition at nucleotide 1298 (A 1298C), leading to a glutamate to alanine substitution in the MTHFR protein, and resulting finally in a 40% reduction in the activity of the enzyme (8). In contrast to C 677T, where homozygosity (TT) results in significant increase in total plasma homocysteine levels, homocysteine concentrations do not appear significantly elevated with the 1298CC genotype(8, 9). Although the presence of MTHFR mutations is significantly more common in miscarried embryos(10), current evidence fails to support an association between these polymorphisms and increased risk for recurrent miscarriage (11-13).Deficiencies of the natural anticoagolant protein C, S and antithrombin occur much less than1% to 2% of the population. Anticoagolant protein deficiencies increased the risk of fetal loss in most, but not all of the limited number of studies.

The EPCOT study showed that the risk for stillbirth (but not miscarriage) is highest in women with combined thrombophilic defects (14). Inevitably, combination of even minor thrombophilic mutations has been studied in association to recurrent miscarriage, with heterogeneous and inconsistent results (11, 12, 15).

## 2. Objectives

In the present study, we compared the frequency of FII G20210A and FVL polymorphisms in a series of patients with three or more consecutive miscarriages with control group. Also we compared activated protein C resistance (APCR), protein C and protein S between the two groups.

## 3. Materials and Methods

### 3.1. Patients and Controls

In this case-control cross-sectional study, 90 patients with three or more consecutive miscarriages with the same partner in <20 weeks gestation were randomly selected. Considering a difference between ratio of 9%, α=0.05 and power 80%, 97 patients were calculated in each group using Power SSC software. Due to low financial support we should decrease number of subjects in the control group to 45. One of the controls had missing data and was excluded from the study. Finally, we had 90 patients in the case group and 44 patients in the control group.All of them were investigated in the Homeostasis and Thrombosis Unit of Hematology Research Center, Shiraz, Southern Iran. The study was carried out from April 2011 to April 2012. The study was approved by the local Ethics Committee of Shiraz university of Medical Sciences (Code:2563, Date: March 2008). In Ethical approval we have been in patient privacy. Three or more pregnancy loss was considered as RPL. We obtained medical histories, performed physical examinations, routine laboratory tests, fibrinogen level, endocrinologic examinations, 5 molar urea test for detection of factor XIII deficiency, and immunologic tests for autoantibodies for patients. Exclusion criteria were: anatomic abnormalities, endocrinologic and liver dysfunction, inflammatory pelvic disease, polycystic ovary syndrome, fibrinogen deficiency, antiphospholipid antibodies and factor XIII deficiency. Seven patients were excluded from the study based on the mentioned criteria and 90 patients were eligible for participation.

The control population consisted of 44 age-matched women, with at least one live born children and no history of pregnancy loss. These women were recruited during their attendance to the Gynecology Outpatient Clinic for a routine Pap smear. All participants were from the same ethnical background and gave their informed consent before inclusion to the study. One observer evaluated all patients and controls for participation in the study.

### 3.2. Laboratory Evaluation

Venipuncture was performed on day 8-10 of the menstrual cycle in order to examine thrombophilic factors. Venous blood was collected on 0.129 mol/l trisodium citrate and was centrifuged twice at 2000 g for 15 min at room temperature in order to obtain plasma with relatively few remaining platelets. Plasma was then frozen and stored in small aliquots at – 70C until tested. Ethylene diamineteraacetic acid (EDTA)- anticoagulant samples were used for DNA analysis. EDTA blood was snap-frozen and immediately stored at – 70C. Genomic DNA was prepared from blood samples according to standard methods.

### 3.3. Protein C and S Analysis

Functional activity of protein C and S and APCR were assessed on plasma which was collected on tubes containing 0.129 mol/L trisodium citrate by auto analyzer ILACL 9000 (Italy) and IL Hemocil Kit (Italy). Normal ranges of protein C and protein S activity were considered as 70-140% and 63-135% respectively. Activated protein C resistance more than 2.2 was considered abnormal.

### 3.4. Factor V Leiden Analysis

A 287 bp fragment of the factor V gene containing the base pair 1691 G A was amplified using polymerase chain reaction (PCR) (16). Digestion of the PCR products containing the wild type, heterozygous and homozygous allele with the restriction enzyme MnII results in: 37, 93, 157, 130, 93, 37; and 157, 130 bp fragments respectively.

### 3.5. Prothrombin G20210A Analysis

A 345 bp fragment of the prothrombin gene containing the base pair 20210G A was amplified using PCR (17). Digestion of the PCR products containing the wild type heterozygous and homozygous allele with the restriction enzyme Hind III results in: 345, 322, 23 and 322, 23 bp fragments respectively.

### 3.6. Statistical Analysis

Chi-square or Fisher's exact test were used for comparisons of polymorphism distribution between the groups. Student t-test was used for comparison of quantitative variables between case and control groups. P value < o.o5 was considered statistically significant.

## 4. Results

The mean age of patients (29.21 ± 5.9 years) did not differ from that of the controls (28.75 ± 5.2 years) (P = 0.66).

Four patients (4.4%) and 1 healthy individual (2.3%) of control group had protein C deficiency (P = 0.906). Protein S deficiency was detected in 9 (10%) patients. Protein S deficiency was not found in the control group (OR = 1.11, 95% CI = 1.03-1.19, P = 0.03). The results of screening tests of inherited thrombophilia in case and control groups are shown in Table 1. The mean functional activity of protein C and protein S were not significantly different between case and control groups (P > 0.05). 

There were three homozygous and fifteen heterozygous cases of Factor V Leiden in the patient group. Genotype distribution of FVL was not significantly different between patients and controls (P = 0.801) (Table 2). 

There were no homozygous cases of Prothrombin G20210A in either group. Genotype distribution of Prothrombin G20210A was not significantly different between patients and controls (P = 0.052) (Table 3). 

**Table 1.** Comparison of the results of screening tests of inherited thrombophilia between case and control groups

	Patients (n = 90)	Controls (n = 44)	P value
**Protein C**	109.7 ± 45.3	102.3 ± 42.1	0.366
**Protein S**	91.3 ± 35.4	85.4 ± 33.1	0.356
**APCR**	2.6 ± 0.2	2.5 ± 0.5	0.101

**Table 2.** Distribution of Factor V Leiden in Women With Recurrent Miscarriage and Control Women

Genotype	Patients (n = 90)	Controls (n = 44)	P value
**Homozygous (AA)**	3	0	0.801
**Heterozygous (AG)**	15	6	
**Wild type (GG)**	72	38	

**Table3.** Distribution of Prothrombin G20210A in Women With Recurrent Miscarriage and Control Women

Genotype	Patients (n=90)	Controls (n=44)	P value
**Homozygous (AA)**	0	0	0.052
**Heterozygous (AG)**	2	1	
**Wild type (GG)**	88	43	

## 5. Discussion

We found that two common thrombophilic mutations (Factor V Leiden and Prothrombin G20210A) are not significantly associated with the occurrence of recurrent miscarriage.

In the meta-analyses of Robertson et al. (3) and Rey et al. (17) Factor V Leiden and FII G20210A were the only thrombophilic mutations associated with recurrent miscarriage. The prevalence of Factor II G20210A polymorphism in our patients and control group is similar to the integrated results of Rey et al.(17) Factor V Leiden is less common in our patients (only 3 homozygous cases).

As there is regional and ethnic variation in the distribution of thrombophilic polymorphisms, we compared our rates with reports from the same region. In our study, the frequency of homozygous FVL polymorphism in patients and controls were similar, whereas Foka et al. (18) reported higher prevalence of the Factor V Leiden in Greek women, despite the similar inclusion criteria and ethnic origin. There was no homozygous case for Prothrombin G20210A polymorphism in our sample, which is similar to the 1/150 rate in women from Chicago, IL, USA reported by Coulam et al (11). The rate of homozygosity for Prothrombin G20210A polymorphism in both our patients and controls appears slightly lower than the reported in similar studies in Central European and American populations (10-14% for patients and 0-16% for controls), whereas there are no comparable data from our region. Factor V Leiden and FII G20210A are associated with pregnancy loss in both the first and second trimester (19, 20).

Regarding the association of protein C and S with RPL, , similar to the previous studies (21, 22) we found a significant association of RPL with protein S deficiency and a non significant correlation with protein C deficiency. In our study patients with RPL showed a non significant higher APC resistance in comparison with healthy women.When analyzing the association between RPL and APC resistance it should be emphasized that the APC sensitivity ratio falls progressively throughout normal pregnancy– a change that can occur independently of factor VIII, factor V and protein S levels. Transient APC resistance can be documented during normal gestations, even in women with normal factor V genotype, and APC sensitivity ratios may be further reduced during gestation – potentially explaining the higher prevalence of vascular complications in women with factor V Leiden mutation. Of interest, APC resistance in the absence of factor V Leiden mutation has also been recently associated with pregnancy loss (23, 24). In a prospective case-control study, APC resistance was the most common thrombophilic defect, documented in 39% of 145 women with pregnancy loss compared to only 3% of control group, with about half of the cases attributed to APC resistance without factor V Leiden (OR 1.4, 95% CI:7.0-53. 6, P, 0:0001) (23-25). Autoantibodies such as anti-b2 glycoprotein-1 are one potential mechanism for acquired APC resistance.

Although recent studies demonstrated that factor II G20210A as a solitary defect is not associated with an increased risk for RPL, compared to controls, they cannot rule out the possibility that factor II G20210A mutation is a risk factor for certain types of RPL (26, 27). Indeed, in a recent study from Italy, Martinelli et al (28) have documented that both factor V Leiden and factor II G20210A mutations are associated with intrauterine fetal demise (IUFD). Eleven of the 67 women with late loss (16%) and 13 of the 232 control women (6%) had either the factor V or the prothrombin mutation. The relative risks of late fetal loss in carriers of the factor V and prothrombin mutations were 3.2 (95% CI, 1.0-10.9) and 3.3 (95% CI, 1.1-10.3), respectively. Thus, both the factor V and the prothrombin mutations were associated with an approximate tripling of the risk of late fetal loss.

In the majority of women with inherited thrombophilia pregnancy is uneventful. However, the risks for miscarriage, IUFD, placental abruption and severe intrauterine growth restriction are increased in carriers of thrombophilia. Why certain women with thrombophilia present with gestational vascular complications is still unknown but may be related to a combined effect with another inherited or acquired prothrombotic risk either systemic or operating locally at the placental level (26, 27).

Fibrin deposition and infarction are found in the placentas of women with thrombophilia and poor gestational outcome. Reports on prophylaxis with low-molecular-weight heparin (LMWH) and unfractionated heparin (UFH), with or without aspirin, suggest a benefit in women with gestational vascular complications and previous poor pregnancy outcome (26, 27).

Controlled trails presently underway will hopefully define the role of antithrombotics on gestational outcome in women with thrombophilia (27).

Our study was limited due to small sample size because of low financial support. The strong point of our study was that we conducted a complete screening for detection of inherited thrombophilia in our patients. With estimated power of 80%, we should evaluate more patients and controls. However, power of this study is acceptable and calculated as 70%.

In conclusion, we determined a significant higher frequency of protein S deficiency in patients with RPL compared with healthy controls. But the frequency of protein C deficiency and the frequency of two common thrombophilic mutations (Factor V Leiden and Prothrombin G20210A), were not significantly different between patients with recurrent miscarriage and healthy women. Further studies with larger numbers are recommended for better evaluation of FVL and prothrombin mutation in patients with RPL.
